# Sportive agoraphobia: scale development and validation

**DOI:** 10.3389/fpsyg.2025.1732295

**Published:** 2026-01-05

**Authors:** Murat Celebi, Neslihan Nur Pehlivan, Mustafa Arici, Mert Ayrancı, Mehmet Ismail Tosun, Abdulsamet Efdal, Murat Sarikabak, Milan Markovic

**Affiliations:** 1 Department of Physical Education and Sports, Faculty of Sport Sciences, Bartın University, Bartın, Türkiye; 2 Department of Therapy and Rehabilitation, Health Services Vocational School, Hitit University, Çorum, Türkiye; 3 Department of Recreation, Faculty of Sport Sciences, Hitit University, Çorum, Türkiye; 4 Department of Physical Education and Sport, Faculty of Sport Sciences, Hitit University, Çorum, Türkiye; 5 Department of Environmental Protection Technologies, Health Services Vocational School, Hitit University, Çorum, Türkiye; 6 Faculty of Sport and Physical Education, University of Priština in Kosovska Mitrovica, Leposavić, Serbia

**Keywords:** body image concerns, exercise avoidance, self-presentation theory, social evaluation anxiety, sportive agoraphobia

## Abstract

**Introduction:**

The present study aimed to develop a valid and reliable instrument, the Sportive Agoraphobia Scale (SAS) to measure individuals’ anxiety and avoidance behaviors related to social visibility, body image, and evaluative concerns in physical activity settings.

**Methods:**

Conducted within the general survey model, data were collected from 352 participants in four provinces of Türkiye. The scale development followed an established eight-step framework. An initial pool of 30 items was reduced to 24 based on expert feedback, and exploratory factor analysis (EFA) identified a three-factor structure: (1) Psychosocial Participation Anxiety, (2) Social Evaluation Anxiety, and (3) Body Image–Based Anxiety. Confirmatory factor analysis (CFA) was conducted after removing four items with low factor loadings, resulting in a final 19-item version of the scale.

**Results:**

Fit indices indicated a good model fit [χ^2^/df = 2.688, RMSEA = 0.069, CFI = 0.946, IFI = 0.946, NFI = 0.917]. Internal consistency values were high for the total scale (*α* = 0.95) and across subdimensions (Factor I: α = 0.93, Factor II: α = 0.85, Factor III: α = 0.87).

**Discussion:**

The findings demonstrate that the Sportive Agoraphobia Scale is a psychometrically sound tool for assessing the multidimensional nature of anxiety stemming from social presence, fear of negative evaluation, and appearance-based concerns in exercise environments. The SAS offers practical implications for researchers, sport psychologists, and intervention designers aiming to understand and mitigate psychological anxiety to physical activity participation.

## 1 Introduction

In social life, individuals’ presence in public spaces extends beyond mere physical existence, encompassing complex psychological processes such as social evaluation, visibility, and performance pressure. In this context, agoraphobia is defined as a marked fear or anxiety about situations in which escape might be difficult, or help may not be available, often leading to avoidance of crowded or socially demanding environments (American Psychiatry Association, 2000; Hackmann, 2004). Although agoraphobia has traditionally conceptualized within the broader framework of anxiety and social phobia, recent research indicates that agoraphobic tendencies can manifest across diverse contextual domains. One of the most prominent emerging areas is the realm of sport and physical activity, where concerns about visibility, judgement and performance pressure may similarly evoke avoidance responses (Roderick and Hockin-Boyers, 2024).

Sport serves as a platform where individuals not only exhibit their physical abilities but also present their bodies, emotions, and social identities within the public sphere. With the rise of visual culture and the pervasive influence of social media, athletic participation has increasingly become a site of public observation, evaluation, and interpretation (Lebel and Danylchuk, 2014). Therefore, anxiety experienced in sport settings is not solely limited to performance; it may also be shaped by efforts to preserve self-worth under social visibility, heightened self-awareness, and the influence of perceived social scrutiny. As outlined in Leary and Kowalski (1990) Self-Presentation Theory, this phenomenon reflects the individual’s effort to manage their image in the eyes of others and to create a favorable impression. In sport settings, this self-presentation process can trigger heightened anxiety and avoidance behaviors, particularly as the perception of being evaluated increases.

Within this framework, the concept of “sportive agoraphobia” has begun to emerge in the literature as a multidimensional manifestation of the psychosocial pressure, avoidance behaviors, and visibility-related anxiety individuals experience while engaging in physical activity under public scrutiny. One of the most recent and influential studies to provide a theoretical foundation for this concept is the article by Roderick and Hockin-Boyers (2024), titled Toward a Sportive Agoraphobia of Professional Athletes. This study demonstrates how professional athletes, when training or competing in public environments, are subjected to constant observation and exposure of their bodies, leading over time to the need for emotional labor. Particularly among elite-level athletes, this state of visibility becomes a source of stress that extends beyond physical performance, intruding upon how they manage their emotions and preserve their privacy. By conceptualizing sportive agoraphobia, the article offers the first theoretical framework in this area and directly informs the current scale development study.

However, sportive agoraphobia is not exclusive to elite or professional athletes. An increasing number of individuals experience body image-related social anxiety and fear of being judged in public exercise environments such as gyms or outdoor spaces (Sarıkabak, 2018; Sabiston et al., 2019). In today’s social media landscape, the constant circulation of idealized body images has led individuals to associate sport not only with physical health, but also with appearance and social approval (Holland and Tiggemann, 2016; Grogan, 2021). The prominence of “fit” or “ideal” body representations on visually oriented social media platforms (e.g., Instagram, TikTok) has contributed to individuals’ negative evaluations of their own bodies and intensified processes of social comparison (Fardouly and Vartanian, 2016; Daniels et al., 2020). Among young women and young adults in particular, this phenomenon increases levels of social appearance anxiety and self-objectification, transforming sport from a health-promoting activity into a domain of social performance and validation (Saunders and Eaton, 2018; Prichard et al., 2020). In this context, the transformation of the body into an object not only of physical competence but also of social approval and visibility is critically important for understanding anxiety and avoidance behaviors in sporting environments. The concept of sportive agoraphobia refers to anxiety individuals experience in exercise settings related to bodily visibility and social judgment. This concept can be understood not merely as a form of individual fear, but also as a reflection of the visibility pressure that is reproduced through media and cultural narratives (Fredrickson and Roberts, 1997; Döring et al., 2016). The observation, comparison, and evaluation of an individual’s body in public spaces generate stress on both cognitive and emotional levels; over time, this stress may lead to avoidance of exercise or a preference for engaging in physical activity alone.

Three theoretical frameworks are particularly prominent in explaining these processes. First, Self-Presentation Theory highlights individuals’ desire to create a positive impression in social settings and their fear of being negatively evaluated by others (Leary and Kowalski, 1990). In social contexts where the body is visible such as exercise settings this anxiety intensifies and can negatively affect performance. Second, the Social Evaluative Threat Model posits that when individuals perceive themselves as being evaluated by others, their physiological and psychological stress responses are heightened (Dickerson and Kemeny, 2004). This perspective is particularly valuable in explaining how being in the public eye during exercise can generate physical and emotional tension for individuals. Third, Objectification Theory argues that women are subjected to constant observation and evaluation in society, which heightens self-awareness and fosters a persistent, appearance-focused mode of thinking centered around the question, “How do I look?” (Fredrickson and Roberts, 1997). Such self-focused attention can transform sport from a source of enjoyment or health into a social stage where bodily performance is displayed. When considered together, these three theoretical frameworks suggest that sportive agoraphobia is not solely linked to an individual’s level of anxiety but is also shaped by bodily self-awareness influenced by social norms, cultural visibility, and media exposure. Accordingly, the present study aims to develop and validate a new construct the Sportive Agoraphobia Scale (SAS) designed to measure fear of social visibility and bodily evaluation in physical activity contexts.

When individuals’ social anxieties during participation in physical activity are combined with fears of negative body evaluation, they create fertile ground for the emergence of agoraphobic symptoms within sportive contexts (Gan and Jiang, 2024; Jiang and Wang, 2025).

The first subdimension, ‘Psychosocial Participation Anxiety,’ encompasses individuals’ hesitations about being present in social environments, fears of exclusion, and concerns regarding their social performance competence. This construction is directly related to avoidance behaviors, which are among the core symptoms of agoraphobia (Wittchen et al., 2010). In particular, low perceived self-efficacy and a sense of inadequate social skills can lead individuals to withdraw from physical activity (Niu et al., 2025).

The second subdimension, ‘Social Evaluation Anxiety,’ encompasses individuals’ fears of being observed, criticized, or negatively judged by others. This factor establishes a strong cognitive bridge between social phobia and agoraphobia. The literature emphasizes that fear of social evaluation is a significant contributor to avoidance of exercise, particularly in group settings (Focht and Hausenblas, 2004; Weeks et al., 2008).

The third subdimension, ‘Body Image–Based Anxiety,’ involves fears of not conforming to societal body norms, feelings of shame about one’s body, and discomfort with bodily visibility. This construct tends to be particularly pronounced among women in environments where the body is on display, such as gyms. Moreover, meta-analyses have shown that body image concerns are significantly associated with exercise avoidance behavior (Hausenblas and Fallon, 2006; Grogan, 2021).

In conclusion, this scale aims to contribute to the field both theoretically and practically by measuring not only anxiety related to sport behavior, but also the underlying social, cognitive, and bodily dynamics of that anxiety. In doing so, the concept of sportive agoraphobia is both clearly defined within academic literature and rendered measurable.

## 2 Materials and methods

### 2.1 Research group

In scale development studies, it is recommended that the sample size be at least twice the number of items and preferably up to ten times to ensure adequate levels of reliability and validity (Büyüköztürk, 2024). Based on an extensive review of the literature and related sources, an initial item pool consisting of 30 items was generated and submitted for expert review. In line with the feedback received from experts, 6 items were removed from the scale, resulting in a final version comprising 24 items. The scale form, which also included items assessing participants’ demographic characteristics, was administered to a total of 374 individuals selected through a convenience sampling method (Creswell, 2002) from four different provinces in Türkiye. This sample size met the ten-to-one participant-to-item ratio recommended by Büyüköztürk (2024). Upon reviewing the submitted forms, researchers identified 22 that were incomplete or incorrectly filled out. These forms were excluded from the analyses, and validity and reliability analyses were conducted on the remaining 352 valid responses. Demographic information about the participants included in the study group is presented in Table 1.

**Table 1:** Demographic distribution and physical activity characteristics of the participants.

Variable	Groups	f	%
Gender	Female	217	61.6
	Male	135	38.4
Educational Background	High School	24	6.8
	Associate degree	134	44.9
	Bachelor’s degree	186	52.8
	Graduate degree	8	2.3
Do you exercise regularly?	Yes	92	26.1
	No	92	26.1
	Occasionally/Irregularly	168	47.7
Preferred environment for physical activity	Indoor (e.g., gym, fitness center)	134	38.1
	Outdoor (e.g., park, walking trail)	108	30.7
	At home	55	15.6
	I do not engage in physical activity	55	15.6
Preferred mode of participation in exercise	Alone/Individually	205	58.2
	Group activity/Team sport	94	26.7
	I do not engage in physical activity	53	15.1
Weekly duration of physical activity	Less than 150 min	154	43.8
	150 min or more	123	34.9
	I do not engage in physical activity	75	21.3

An examination of Table 1 reveals that most participants were female (61.6%). Regarding educational background, the highest proportion held a bachelor’s degree (52.8%), followed by those with an associate degree (44.9%), high school diploma (6.8%), and a graduate degree (2.3%). In terms of regular physical activity, 47.7% reported engaging in exercise occasionally or irregularly, 26.1% exercised regularly, and 26.1% did not engage in physical activity at all. With respect to preferred exercise environments, indoor settings (e.g., gyms) were the most preferred (38.1%), followed by outdoor settings (30.7%). Exercising at home and not exercising at all were equally reported (15.6%). Most participants (58.2%) preferred to exercise individually, while 26.7% participated in group or team activities, and 15.1% reported not engaging in any physical activity. Regarding weekly exercise duration, 43.8% exercised for less than 150 min, 34.9% exercised for 150 min or more, and 21.3% reported no physical activity.

### 2.2 Scale development process

To develop a valid and reliable measurement tool for sportive agoraphobia, both Turkish and English literature were reviewed to identify the existing gap in available scales. The scale development process was based on the eight-step model proposed by DeVellis and Thorpe (2021).

In the first stage, based on a comprehensive literature review, the researchers developed an initial item pool consisting of 30 items for the intended scale. In the second stage, the 30 items were submitted for expert review to ensure content validity. Feedback was obtained from four experts, two language specialists and two subject matter experts. In the third stage, based on the feedback received, six items were removed, reducing the scale to 24 items. In the fourth stage, the remaining 24 items were randomly ordered within the scale and evaluated using a five-point Likert-type response format: Strongly Agree (5), Agree (4), Neutral (3), Disagree (2), Strongly Disagree (1). The first draft of the scale was then prepared along with its administration instructions. In the fifth stage, a pilot study was conducted with 144 participants selected from the target population, approximately six times the number of items on the scale. In the sixth stage, item-total correlations were calculated to assess the relationship between each item and the overall scale score. In the seventh stage, validity and reliability analyses were performed. As a result of exploratory factor analysis (EFA), items that loaded on multiple factors or did not align with the underlying structure of the scale were removed. Initially consisting of 24 items across three factors, the scale was refined to a final structure comprising 19 items and three factors. In the EFA stage, items with factor loadings below 0.40 or cross-loadings above 0.32 were identified as candidates for removal. In the CFA stage, items with substantially low standardized factor loadings (typically below 0.60) or those that weakened the overall model fit were removed. Items with loadings close to 0.70 but demonstrating strong theoretical relevance and contributing adequately to the latent construct were retained, in accordance with recommendations by Tabachnick and Fidell (2019) and Kline (2023). To assess the internal consistency of the items, both Cronbach’s alpha and McDonald’s omega coefficients were calculated. Further details of this stage are elaborated under the sections on exploration and confirmatory factor analyses. In the eighth and final stage, the finalized version of the Sportive Agoraphobia Scale was constructed, consisting of 19 items grouped under three subdimensions: 10 items under “Psychosocial Participation Anxiety,” 4 items under “Social Evaluation Anxiety,” and 5 items under “Body Image–Based Anxiety.” The final form of the scale is designed to measure participants’ levels of sportive agoraphobia.

## 3 Results

### 3.1 Exploratory factor analysis (EFA)

Prior to conducting a Principal Component Analysis (PCA) to examine the factor structure of the Sportive Agoraphobia Scale, a scree plot of eigenvalues greater than 1 and the variance explained by each factor were analyzed. The results indicated the presence of three factors with eigenvalues exceeding 1. Specifically, the first factor had an eigenvalue of 12.16 and accounted for 50.69% of the total variance. The second factor had an eigenvalue of 1.56, explaining 6.52% of the variance, while the third factor had an eigenvalue of 1.05, contributing 4.38% to the total variance. Collectively, these three factors explained 61.60% of the total variance. Although the analysis initially supported a three-factor structure, a subsequent Principal Component Analysis with oblimin rotation revealed that one item displayed cross-loadings and was therefore removed from the scale. The remaining 23 items were reanalyzed using the full sample. Following this revision, the 23 items grouped under three factors were found to explain 63.54% of the total variance. The Kaiser-Meyer-Olkin (KMO) measure of sampling adequacy was calculated as 0.962, well above the recommended threshold of 0.60, indicating suitability for factor analysis. Additionally, Bartlett’s Test of Sphericity yielded a statistically significant result [χ^2^ = 5848.314, *p* < 0.05], confirming the appropriateness of the data set for factor analysis (Bartlett, 1954).

**Figure 1:**
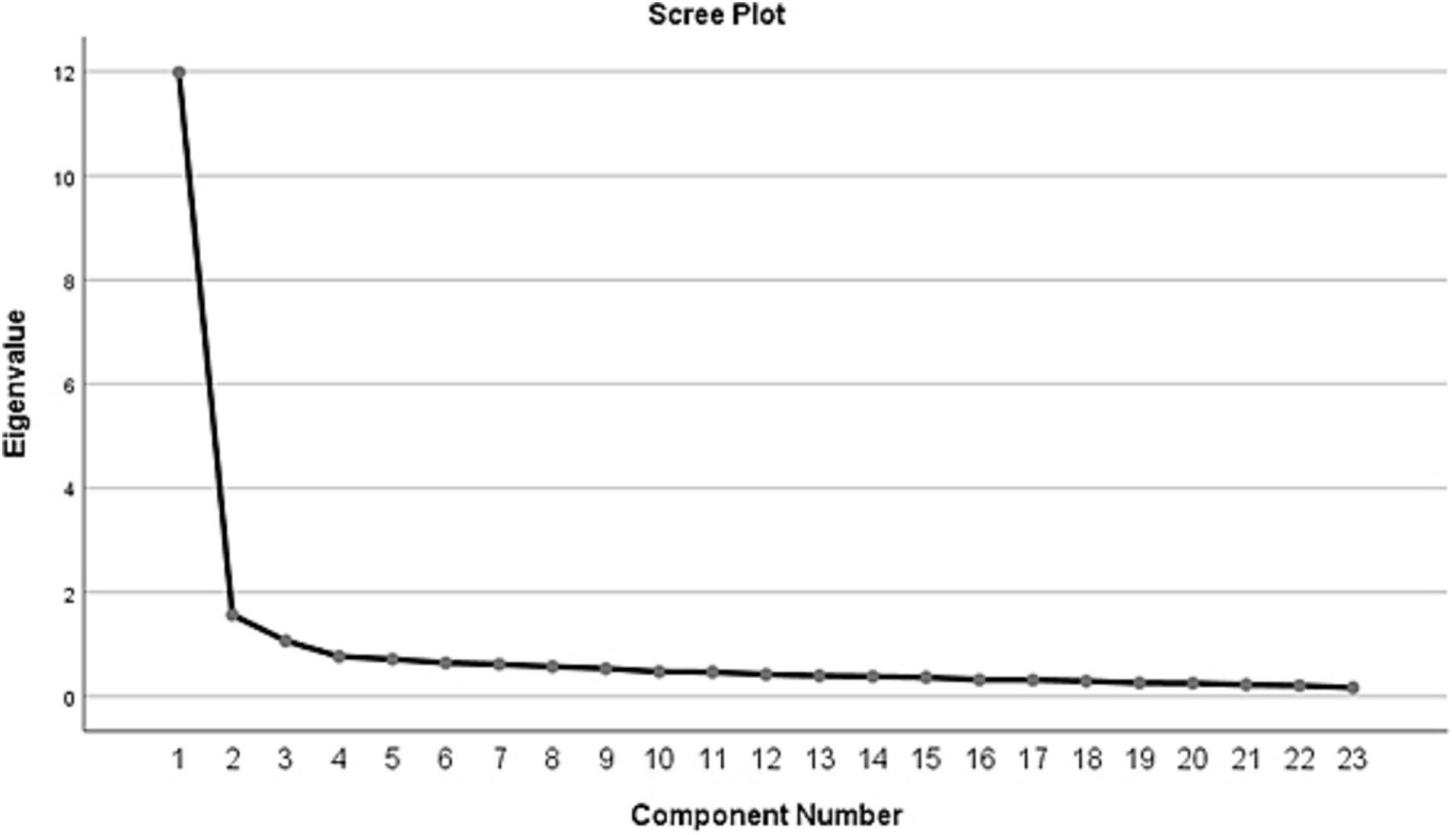
Scree plot output.

The findings related to the factor analyses of the final 23 items included in the scale are presented in Figure 1 and Table 2.

**Table 2:** Exploratory factor analysis results of the Sportive Agoraphobia Scale.

Factor	Item	Communality	Rotated loadings		
			Factor I	Factor II	Factor III
Factor 1 Psychosocial participation anxiety/barriers	22	0.575	0.819		
	1	0.634	0.794		
	16	0.643	0.783		
	17	0.679	0.749		
	23	0.711	0.738		
	19	0.707	0.725		
	20	0.691	0.700		
	18	0.722	0.662		
	21	0.656	0.616		
	2	0.617	0.519		
Factor 2 Social evaluation anxiety	4	0.604		0.798	
	5	0.713		0.640	
	6	0.590		0.620	
	3	0.609		0.544	
	7	0.683		0.474	
Factor 3 Body image–based anxiety	13	0.710			−0.801
	11	0.633			−0.628
	10	0.482			−0.590
	14	0.608			−0.584
	15	0.510			−0.580
	8	0.638			−0.553
	9	0.660			−0.489
	12	0.540			−0.486

An examination of Figure 1 indicates that there are three components with eigenvalues greater than 1. Since the number of factors is typically determined by the number of components with eigenvalues exceeding 1, it was concluded that the scale possesses a three-factor structure. The first factor accounts for 52.096% of the total variance in the scale, the second factor explains 6.811%, and the third factor contributes 4.634%. Collectively, these three factors explain 63.541% of the total variance. This proportion is consistent with the commonly accepted threshold of 60% or higher in factor analyses within the social sciences, thereby supporting the structural validity of the scale (Fabrigar et al., 1999).

As a result of the exploratory factor analysis, the scale demonstrated a three-factor structure. In the structure obtained through oblimin rotation, the first factor was labeled “Psychosocial Participation Anxiety” and comprised 10 items (Items 22, 1, 16, 17, 23, 19, 20, 18, 21, and 2). The rotated factor loadings for these items ranged from 0.519 to 0.819, while their communalities ranged from 0.575 to 0.722. The second factor, named “Social Evaluation Anxiety,” consisted of 5 items (Items 4, 5, 6, 3, and 7), with factor loadings ranging from 0.474 to 0.798 and communalities between 0.590 and 0.713. The third factor, defined as “Body Image –Based Anxiety,” included 8 items (Items 13, 11, 10, 14, 15, 8, 9, and 12). The factor loadings in this dimension ranged from −0.486 to −0.801, and communalities varied between 0.482 and 0.710. This structure indicates that all items have statistically significant and adequate factor loadings and that the items are grouped meaningfully under their respective factors in terms of content. Accordingly, it can be concluded that the scale possesses a three-dimensional structure and that the items have sufficient explanatory power in representing their corresponding factors.

### 3.2 Confirmatory factor analysis (CFA)

The model fit of the factor structure identified through Exploratory Factor Analysis (EFA) was tested using Confirmatory Factor Analysis (CFA). The adequacy of the model was evaluated using several fit indices, including the Root Mean Square Error of Approximation (RMSEA), Normed Fit Index (NFI), Comparative Fit Index (CFI), and Incremental Fit Index (IFI). During the CFA process, standardized factor loadings for each item were examined in relation to their corresponding factors in order to improve model fit and ensure structural validity. Based on this examination, four items (Items 4, 10, 14, and 15) with standardized loadings below 0.70 were removed from the model, as such low loadings indicate that the items do not adequately represent their respective constructs (Tabachnick and Fidell, 2019; Kline, 2023). Following this modification, the model was retested, and higher fit indices were obtained (Table 3).

**Table 3:** Fit indices for the three-factor first-order model of the scale.

Model	*X* ^2^	*df*	*p*	*X*^2^/*df*	RMSEA	CFI	IFI
Three-factor first-order model	400.510	149	0.000	2.688	0.069	0.946	0.946

As a result of the Confirmatory Factor Analysis (CFA), the three-factor model proposed through Exploratory Factor Analysis (EFA) was tested. In the initial analysis, four items with factor loadings below 0.70 were removed from the model. Examination of the fit indices for the unmodified model yielded the following results: [*χ*^2^ = 400.510, df = 149, *p* < 0.001, *χ*^2^/df = 2.688, RMSEA = 0.069, CFI = 0.946, IFI = 0.946, NFI = 0.917]. The fact that the *χ*^2^/df ratio is below 3 and the other fit indices exceed 0.90 indicates that the model demonstrates a good fit with the data (Şimşek, 2020; Meydan and Şeşen, 2011) Moreover, the RMSEA value being below 0.08 further supports the acceptability of the model’s structure (Sümer, 2000). Following this modification, the model was retested, and higher fit indices were obtained.

An examination of Table 4 reveals that the t and R^2^ values obtained from the confirmatory factor analysis indicate a high level of measurement strength for the scale. Within the first factor, Items 18, 19, and 23 demonstrated the highest explanatory power, with R^2^ values of 0.84, 0.82, and 0.83 respectively, suggesting that these items best represent the underlying construct. Similarly, in the second factor, Item 7 (0.83) and Item 3 (0.75) exhibited the strongest explanatory power. In the third factor, Items 9 and 8 showed high contributions, with R^2^ values of 0.83 and 0.79, respectively.

**Table 4:** t and R^2^ values of items based on confirmatory factor analysis (CFA) results.

Items	t	R^2^	Items	t	R^2^	Items	t	R^2^
22	11.58	0.67	5	13.83	0.82	13	13.52	0.73
1	11.60	0.67	6	13.75	0.68	11	13.68	0.74
16	12.81	0.75	7	17.83	0.83	9	15.37	0.83
17	13.57	0.80	3	15.52	0.75	12	13.48	0.73
23	13.99	0.83				8	14.67	0.79
19	13.87	0.82						
20	13.80	0.82						
18	14.19	0.84						
21	13.33	0.78						
2	11.99	0.69						

**Figure 2:**
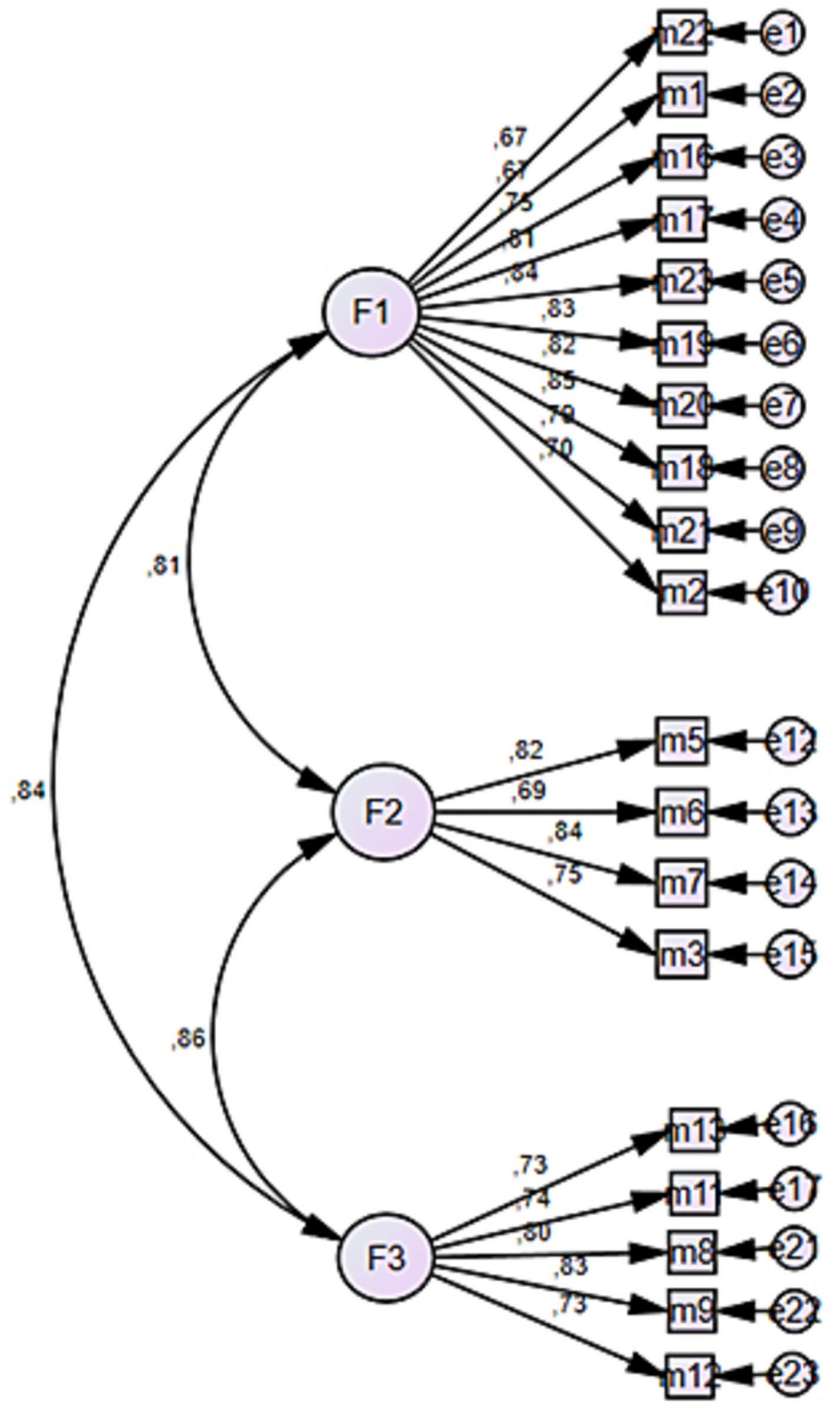
Three-factor confirmatory factor analysis model.

Furthermore, all t-values were found to be statistically significant, and R^2^ values ranged from 0.66 to 0.84, indicating that the model possesses sufficient and satisfactory validity. These findings provide strong support for the structure identified through exploratory factor analysis, confirming its consistency within the confirmatory framework (Figure 2).

As a result of the Confirmatory Factor Analysis (CFA), the three-factor model proposed through Exploratory Factor Analysis (EFA) was tested. In the initial analysis, four items with factor loadings below 0.70 were removed from the model. The fit indices for the unmodified model were as follows: [*χ*^2^ = 400.510, df = 149, *p* < 0.000, χ^2^/df = 2.688, RMSEA = 0.069, CFI = 0.946, IFI = 0.946, NFI = 0.917]. The χ^2^/df ratio being below 3 and the other fit indices exceeding 0.90 indicate that the model demonstrates a good level of fit with the data (Collier, 2020). Moreover, the RMSEA value being below 0.08 further supports the acceptability of the model structure. In light of these findings, it can be concluded that the three-factor structure is both theoretically supported and empirically confirmed.

To strengthen the evidence for construct validity, Composite Reliability (CR), Average Variance Extracted (AVE), and inter-factor correlations were calculated for each dimension of the SAS. The CR values exceeded the recommended threshold of 0.70, and AVE values were above the acceptable limit of 0.50, indicating adequate convergent validity. Additionally, inter-factor correlations remained below 0.90, providing support for discriminant validity. All CR and AVE computations were conducted using the online calculation tool developed by Aydogdu (2023), following the criteria proposed by Fornell and Larcker (1981) (Table 5).

**Table 5:** Construct validity indicators for the three-factor structure of the Sportive Agoraphobia Scale.

Factor	CR	AVE	F1	F2	F3
F1 - Psychosocial participation anxiety	0.937	0.602	1	0.811	0.845
F2 - Social evaluation anxiety	0.858	0.604	0.811	1	0.855
F3 - Body image-based anxiety	0.877	0.588	0.845	0.855	1

### 3.3 Reliability analyses

To assess the internal consistency of the 19-item, five-point Likert-type scale, Cronbach’s alpha coefficient was calculated. After determining the overall Cronbach’s alpha and McDonald’s omega coefficients for the entire scale, factor analysis was conducted, and separate reliability coefficients were calculated for each factor. Table 6 presents the reliability coefficients calculated for the total scale and its subdimensions.

An examination of the Cronbach’s alpha (*α*) and McDonald’s omega (*ω*) internal consistency coefficients presented in Table 6 reveals that both the overall scale and its subdimensions exhibit high levels of internal consistency. For Factor I, both α and ω were calculated as 0.93, indicating a highly reliable structure. The internal consistency coefficients for Factors II and III were 0.85 and 0.87, respectively, suggesting that both factors demonstrate acceptable and sufficient reliability. Furthermore, the overall α and ω values for the entire scale were 0.95, signifying excellent internal consistency. These findings suggest that the developed scale reliably reflects the construct it aims to measure and can be confidently utilized in future research (Table 6).

**Table 6:** Cronbach’s alpha (α) and McDonald’s omega (ω) internal consistency coefficients.

Factors	Number of items	α	*McDonald* ω
*Factor I	10	0.93	0.93
*Factor II	4	0.85	0.85
*Factor III	5	0.87	0.87
*SAS Total	19	0.95	0.95

## 4 Discussion and conclusion

This study established a 19-item, three-factor structure for the Sportive Agoraphobia Scale (SAS), demonstrating strong validity and reliability across all analyses. In the context of sport, psychosocial factors such as social visibility, body image, and fear of social judgment are gaining increasing importance in understanding individuals’ participation in physical activity. In this regard, the Sportive Agoraphobia Scale (SAS), developed through this study, offers a psychometrically robust instrument for multidimensionally assessing individuals’ anxiety and avoidance tendencies when engaging in physical activity in social settings. As a result of the study, a structure consisting of 19 items and three factors was established. The first factor, Psychosocial Participation Anxiety, includes 10 items and accounts for 52.096% of the total variance. This dimension reflects individuals’ discomfort in social environments, fear of exclusion, and concerns about social performance. The Social Evaluation Anxiety dimension, composed of 4 items, explains 6.811% of the variance, while the Body Image–Based Anxiety factor contributes 4.634% with 5 items. The total variance explained by the scale is 63.541%, which exceeds the commonly accepted threshold of 60% for multifactorial scales (Fabrigar et al., 1999). This result indicates that the scale possesses an adequate level of structural validity.

The first factor, Psychosocial Participation Anxiety, encompasses individuals’ discomfort in social settings, fear of exclusion, and concerns related to social performance. The Social Evaluation Anxiety dimension reflects fears of being observed and negatively judged by others, while the Body Image–Based Anxiety factor pertains to dissatisfaction with body image, feelings of shame, and discomfort with visibility. Together, these dimensions offer a comprehensive framework that distinguishes between different levels of anxiety rooted in social evaluation and visibility. The three-factor structure proposed was tested through Confirmatory Factor Analysis (CFA), and the resulting fit indices (χ^2^/df = 2.688, RMSEA = 0.069, CFI = 0.946, IFI = 0.946, NFI = 0.917) indicated that the model demonstrated a high degree of fit with the data (Kline, 2023). Moreover, all item t-values were found to be statistically significant, and the explanatory power (R^2^) values ranged from 0.66 to 0.84, further supporting the reliability of the measurement model.

As a result of the reliability analyses, the internal consistency coefficients of the scale were found to be high both at the overall level (*α* = 0.95, *ω* = 0.95) and for each subdimension (F1 = 0.93, F2 = 0.85, F3 = 0.87). These values indicate that the scale can be used reliably in both research and practical applications. Furthermore, when compared with other instruments measuring similar constructs—such as the Social Exercise and Anxiety Measure (SEAM; Levinson et al., 2013) the SAS appears to fill a unique gap in the literature by simultaneously assessing visibility, social threat, and body image components.

This study offers a unique contribution to literature by operationalizing the concept of sportive agoraphobia at the empirical level for the first time. The findings indicate that the emergence of sportive agoraphobia as a three-dimensional construct is consistent with theoretical expectations in social psychology literature. The emergence of sportive agoraphobia as a three-dimensional construct aligns closely with core theoretical frameworks in social psychology. According to Self-Presentation Theory (Leary and Kowalski, 1990), individuals experience heightened anxiety when they fear that their self-worth may be negatively evaluated in social contexts. Similarly, the Social Evaluative Threat Model (Dickerson and Kemeny, 2004) emphasizes that being observed or judged by others triggers strong cognitive and physiological stress responses. Together, these frameworks explain the mechanisms underlying both the “psychosocial participation anxiety” and “social evaluation anxiety” dimensions of the SAS. Moreover, Objectification Theory (Fredrickson and Roberts, 1997) provides a basis for the “body image–based anxiety” factor, suggesting that increased self-consciousness and perceived surveillance intensify anxiety and avoidance tendencies in exercise environments. Collectively, these theoretical perspectives support the multidimensional structure of sportive agoraphobia and clarify how visibility, evaluation, and comparison processes shape individuals’ emotional responses in sport settings.

In previous studies (Roderick and Hockin-Boyers, 2024), the concept of sportive agoraphobia was addressed solely at a conceptual level, primarily focusing on the experiences of professional athletes under the pressure of public visibility. However, the SAS developed in the present study demonstrates that anxiety related to social visibility is also prevalent in the general population—particularly among women and younger individuals. Although the present study did not include a statistical comparison of SAS scores across genders, prior research consistently shows that women report higher levels of appearance-related anxiety, body dissatisfaction, and social evaluative concerns in exercise environments (Sabiston et al., 2019; Macila et al., 2024). These established gender-based tendencies suggest that visibility-related anxiety may be more pronounced among women, highlighting the value of future research investigating gender differences using the SAS in broader and more diverse samples. Similarly, the concept of social physique anxiety developed by Focht and Hausenblas (2004) closely aligns with the “body image–based anxiety” factor identified in this study. Accordingly, the SAS extends this body of literature by enabling the multidimensional assessment of social evaluation and avoidance behaviors. In doing so, the concept of sportive agoraphobia is redefined not as a phenomenon limited to elite athletes, but as a psychosocial construct observable across all levels of social participation.

The findings of this study indicate that sportive agoraphobia is not only a theoretical construct but also holds significant practical implications. The Sportive Agoraphobia Scale (SAS) can be utilized by sport psychologists, physical education teachers, group trainers, and clinical counselors to screen individuals prior to their participation in exercise environments. Particularly for women, the scale offers opportunities for early intervention in cases where body-related anxiety is high. From a social psychological perspective, the SAS serves not only as a diagnostic tool but also as a guide for structuring intervention processes. Group-based intervention programs that target self-efficacy and the need for social approval (e.g., exercise guidance aimed at enhancing self-efficacy or body awareness workshops) may be effective in reducing symptoms of sportive agoraphobia. For instance, findings indicating that self-compassion and mindfulness-based approaches can reduce anxiety under conditions of heightened visibility (Przezdziecki et al., 2013; Harwood and Kocovski, 2017) could inform future intervention efforts in this area. In addition, SAS may guide the design of clinical or preventive interventions targeting anxiety-related exercise avoidance. Thus, the SAS becomes a tool not only for assessment but also for psychosocial intervention design. It may be especially useful for identifying social avoidance behaviors that emerge in sport settings among adolescents and young adults. Additionally, it can serve as an effective resource in planning motivational counseling strategies for individuals with low participation in group exercise contexts. However, it should be acknowledged that the sample in this study consisted predominantly of female participants (61.6%) and individuals with a university-level education. This demographic distribution may limit the generalizability of the findings to broader populations. Future studies involving more heterogeneous samples recommend enhancing the scale’s applicability across diverse demographic groups.

The Sportive Agoraphobia Scale (SAS) developed in this study, offers a valuable tool for identifying psychological anxiety-related psychological barriers to physical activity by addressing social visibility, body image, and social evaluation anxiety through an integrative approach. With its high psychometric properties, the scale is well-suited for widespread use in both academic research and applied field settings. By measuring the effects of visibility-related anxiety within sport environments, the SAS is expected to contribute a unique and meaningful perspective to the sport psychology literature. Future research should aim to test the cross-cultural validity of the SAS and examine the longitudinal progression of sportive agoraphobic symptoms over time. Additionally, forthcoming studies may investigate the impact of social media–driven visibility culture and gender roles on sportive agoraphobia from a longitudinal perspective. Such inquiries would provide valuable insights into the cross-cultural applicability of both Objectification Theory and the Social Evaluative Threat Model. Moreover, future research should explore the relationship between sportive agoraphobia and variables such as sport identity and sense of belonging, thereby enriching our understanding of the construct’s social identity dimensions. Intervention studies—such as group programs designed to enhance self-efficacy could assess their effects on SAS scores. Lastly, the concurrent validity of the SAS should be examined through comparisons with similar instruments, further reinforcing the scale’s psychometric strength. At the conclusion of the study, the 19-item Sportive Agoraphobia Scale and its corresponding subscale items are presented in Supplementary material 1.

## Data Availability

The raw data supporting the conclusions of this article will be made available by the authors, without undue reservation.
