# Supplementary material for: Sportive agoraphobia: scale development and validation
Source: Front Psychol. 2026 Jan 5;16:1732295. doi: 10.3389/fpsyg.2025.1732295 (PMC12813173; doi:10.3389/fpsyg.2025.1732295)
Supplement: Supplementary file 1 [file Table_1.pdf]

## Supplementary Material

### **S1.** Sportive Agoraphobia Scale (SAS)

| The Sportive Agoraphobia Scale (SAS) is a measurement tool developed to assess the levels of anxiety, discomfort, and apprehension individuals may experience while engaging in physical activity in social settings. This form has been created to help us understand your thoughts and feelings related to exercising. Please read each item carefully and indicate the degree to which you agree with each statement. While responding, consider how you feel—or might feel—when participating in physical activity in social environments. Please rate each item using the scale below: 1 (Strongly Disagree) to 5 (Strongly Agree). There are no right or wrong answers; what matters is that you provide honest and sincere responses that reflect your personal views. Please answer all items completely. | | | Strongly Disagree | Disagree | Neutral | Agree | Strongly Agree |
|---|---|---|---|---|---|---|---|
| Sportive Agoraphobia Scale (SAS) | **1** | I feel uncomfortable when I think others are watching me while I exercise. | 1 | 2 | 3 | 4 | 5 |
| | **2** | I worry that my movements may appear incorrect during exercise. | 1 | 2 | 3 | 4 | 5 |
| | **3** | I worry that my performance will be perceived as inadequate in exercise settings. | 1 | 2 | 3 | 4 | 5 |
| | **4** | I focus too much on what others think of me while exercising. | 1 | 2 | 3 | 4 | 5 |
| | **5** | I feel pressure to "look right" while exercising. | 1 | 2 | 3 | 4 | 5 |
| | **6** | I feel tense in exercise settings because I think others are judging me. | 1 | 2 | 3 | 4 | 5 |
| | **7** | I feel that my body is physically inferior to others during exercise. | 1 | 2 | 3 | 4 | 5 |
| | **8** | I compare my physical appearance to others who are exercising. | 1 | 2 | 3 | 4 | 5 |
| | **9** | I believe I may receive negative evaluations about my body while exercising. | 1 | 2 | 3 | 4 | 5 |
| | **10** | I avoid drawing attention to my appearance during exercise. | 1 | 2 | 3 | 4 | 5 |
| | **11** | I believe that physical appearance is important for social acceptance while exercising. | 1 | 2 | 3 | 4 | 5 |

| | | | | | | | |
|---|---|---|---|---|---|---|---|
| | **12** | I feel uncomfortable exercising in crowded environments. | 1 | 2 | 3 | 4 | 5 |
| | **13** | Exercising in open/public spaces makes me feel uneasy. | 1 | 2 | 3 | 4 | 5 |
| | **14** | The presence of others in gyms makes me feel nervous. | 1 | 2 | 3 | 4 | 5 |
| | **15** | I find it difficult to start exercising in places where people are present. | 1 | 2 | 3 | 4 | 5 |
| | **16** | Exercising in social environments is emotionally challenging for me. | 1 | 2 | 3 | 4 | 5 |
| | **17** | The idea of being in a social setting prevents me from engaging in exercise. | 1 | 2 | 3 | 4 | 5 |
| | **18** | I prefer exercising alone rather than in front of others. | 1 | 2 | 3 | 4 | 5 |
| | **19** | Being in the same space as others while exercising causes me stress. | 1 | 2 | 3 | 4 | 5 |

**Factor Structure:**

The *Sportive Agoraphobia Scale (SAS)* consists of 19 items grouped under three dimensions:
(1) *Psychosocial Participation Anxiety* (10 items: 1, 2, 12–19) – reflects individuals' discomfort, fear of exclusion, and avoidance tendencies in social exercise environments;
(2) *Social Evaluation Anxiety* (4 items: 3–6) – represents the fear of being observed, criticized, or negatively evaluated by others during physical activity;
(3) *Body Image–Based Anxiety* (5 items: 7–11) – captures concerns about body appearance, social comparison, and visibility-related discomfort.
